# Polymeric nanobiotics as a novel treatment for mycobacterial infections

**DOI:** 10.1016/j.jconrel.2019.10.009

**Published:** 2019-11-28

**Authors:** Iris L. Batalha, Audrey Bernut, Mark Schiebler, Myriam M. Ouberai, Charlotte Passemar, Catherine Klapholz, Sonja Kinna, Sarah Michel, Kasim Sader, Pablo Castro-Hartmann, Stephen A. Renshaw, Mark E. Welland, R. Andres Floto

**Affiliations:** a Nanoscience Centre, Department of Engineering, University of Cambridge, 11 J.J. Thomson Avenue, Cambridge, CB3 0FF, United Kingdom; b Molecular Immunity Unit, Department of Medicine, University of Cambridge, Francis Crick Avenue, Cambridge Biomedical Campus, Cambridge, CB2 0QH, United Kingdom; c Dept. of Infection, Immunity & Cardiovascular Disease, Bateson Centre, University of Sheffield, Firth Court, Western Bank, Sheffield, S10 2TN, United Kingdom; d Medical School, University of Sheffield, Sheffield, S10 2RX, United Kingdom; e Cambridge CryoEM Pharmaceutical Consortium, Thermo Fisher Scientific, Nanoscience Centre, Department of Engineering, University of Cambridge, 11 J.J. Thomson Avenue, Cambridge, CB3 0FF, United Kingdom; f Cambridge Centre for Lung Infection, Royal Papworth Hospital, Cambridge, CB23 3RE, United Kingdom

**Keywords:** *Mycobacterium tuberculosis*, Polymer-drug conjugate, Antibiotic, Nanoparticles, Zebrafish, Isoniazid, Clofazimine

## Abstract

*Mycobacterium tuberculosis* (*Mtb*) remains a major challenge to global health, made worse by the spread of multi-drug resistance. Currently, the efficacy and safety of treatment is limited by difficulties in achieving and sustaining adequate tissue antibiotic concentrations while limiting systemic drug exposure to tolerable levels. Here we show that nanoparticles generated from a polymer-antibiotic conjugate (‘nanobiotics’) deliver sustained release of active drug upon hydrolysis in acidic environments, found within *Mtb*-infected macrophages and granulomas, and can, by encapsulation of a second antibiotic, provide a mechanism of synchronous drug delivery. Nanobiotics are avidly taken up by infected macrophages, enhance killing of intracellular *Mtb*, and are efficiently delivered to granulomas and extracellular mycobacterial cords *in vivo* in an infected zebrafish model. We demonstrate that isoniazid (INH)-derived nanobiotics, alone or with additional encapsulation of clofazimine (CFZ), enhance killing of mycobacteria *in vitro* and in infected zebrafish, supporting the use of nanobiotics for *Mtb* therapy and indicating that nanoparticles generated from polymer-small molecule conjugates might provide a more general solution to delivering co-ordinated combination chemotherapy.

## Graphical abstract

**Figure:**
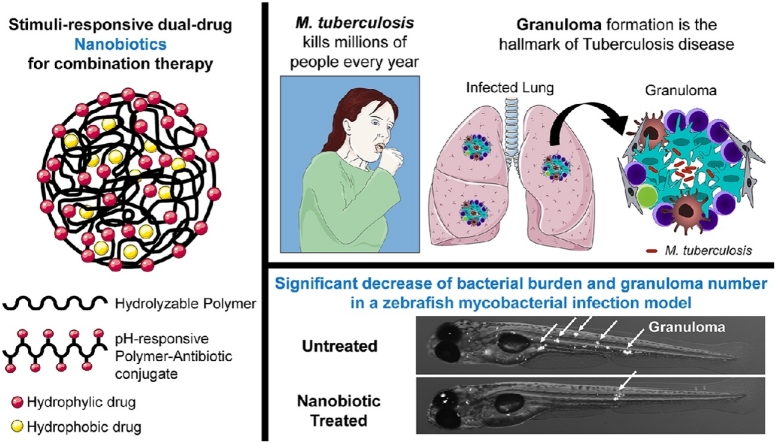

## 1 Introduction

Over 1.6 million deaths annually are caused by *Mtb* infection [1]. Existing antibiotic regimens for *Mtb* infection require long durations of therapy with multiple drugs and are associated with significant side effects (due to systemic exposure), contributing to poor adherence and treatment failure [2].

One of the major difficulties in treating tuberculosis is that *Mtb* can survive both intracellularly within macrophages and extracellularly within granulomas; environments where conventional drug delivery is compromised. Bacteria are therefore exposed to sub-lethal concentrations of antibiotics, permitting firstly the development of phenotypic drug tolerance and eventually the acquisition of drug resistance mutations [2].

Due to the scarcity of new drugs against *Mtb* and thus limited therapeutic options for drug-resistant *Mtb*, increased efforts have been put on the development of improved formulations and delivery systems for existing antibiotic regimens [3].

In the last two decades, the application of polymer-drug conjugation to drug delivery has increased noticeably, offering advantages including enhanced drug solubilization, reduced immunogenicity, controlled delivery, increased efficacy, and improved pharmacokinetics. However, most polymer-small molecule drug conjugates have to date used non-biodegradable polymer carriers, such as polyethylene glycol (PEG), that constrains polymer size below the molecular cut-off of ∼40 kDa required for renal elimination [4]. Alternatively, hydrolysable hydrophobic polyesters, such as polycaprolactone (PCL) and poly(lactide-co-glycolide) (PLGA), widely employed in FDA-approved devices, present limited functionality for drug conjugation [5] and are used to physically entrap drugs within nanoparticulate carriers [6]. However, many anti-tuberculosis drugs are highly water soluble, making them easily leached out from the nanocarriers during fabrication and more prone to burst release in systemic circulation [[7], [8], [9], [10]]. Polyketals, which in contrast with polyesters yield pH neutral hydrolysis products, have also recently been explored as new class of acid responsive and biodegradable polymers suitable for drug conjugation [11].

Isoniazid (INH) is a potent antibiotic universally used as a first-line treatment of tuberculosis, either as part of combination therapy to treat the active disease, or often used as monotherapy in cases of latent tuberculosis infection. Despite its high activity against *Mtb*, INH is rapidly egested and highly toxic, prompting the development of delivery systems aiming for targeted and controlled release of INH [12,13]. A few polymers have been explored for INH conjugation, including natural polymers, such as gelatin [14] and chitosan [13], and synthetic polymers, such as PLGA [15]. However, these systems involve further chemical modifications of the polymers in order to introduce functional groups amenable for drug conjugation. Berezin and Skorik prepared chitosan-INH conjugates using two different synthetic routes, either by modifying chitosan with acrylic acid or epichlorohydrin, before INH conjugation. Modified chitosan polymers presented lower biodegradability, and either similar (for *N*-(2-carboxyethyl)chitosan INH conjugates) or higher (for *N*-(3-chloro-2-hydroxypropyl)chitosan INH conjugates) minimum inhibitory concentrations compared to free drug, possibly due to incomplete cleavage of INH from the polymer [13]. In a different study, Huang and co-workers used an INH conjugated star PLGA to fabricate a composite scaffold with β-tricalcium phosphate to treat bone tuberculosis. The process involved esterification of the PLGA and 4-carboxybenzaldehyde prior to drug conjugation. They have produced a 4-arm PLGA-INH conjugate instead of a linear polymer in order to achieve suitable drug loading capacity [15].

Another important aspect for combination therapy is the ability to co-deliver multiple drugs to the target sites. Manca and co-workers prepared microparticles of gelatin-INH conjugates with encapsulated rifampicin by spray drying technique. INH-derivatized gelatin was prepared by heterogenous reaction of amidation, yielding an amide bond between the terminal acyl chloride group of gelatine and the hydrazide group of INH. They have shown good nebulization efficiency, cell internalization, and low cytotoxicity, but they have not reported the therapeutic efficacy of the conjugates [14].

As a response to these challenges, we have developed a polymeric nanoparticulate drug delivery system, using simple, fast and scalable processes, where antibiotics are covalently incorporated into a polymer chain, through a hydrolysable bond, creating ‘nanobiotics’.

**Fig. 1:**
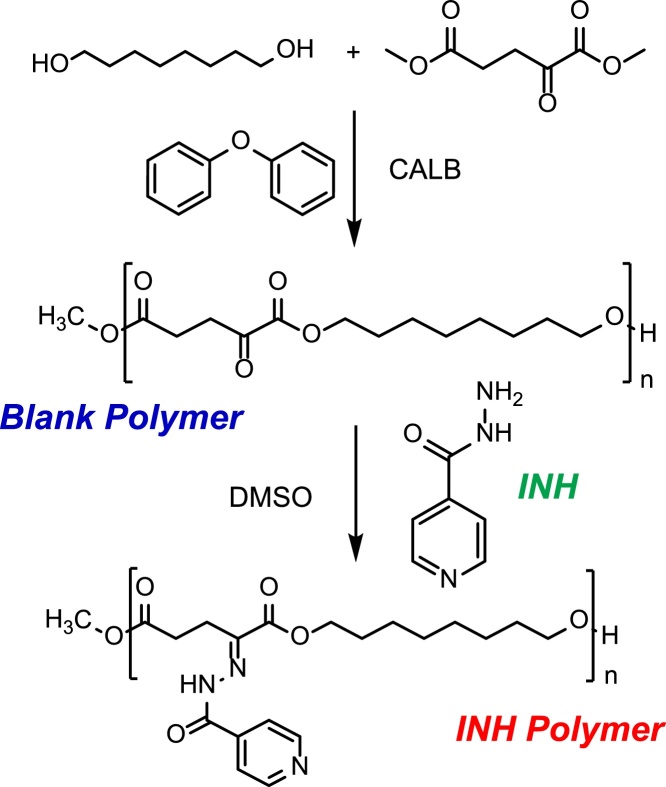
Synthesis of an α-keto polyester by (trans)esterification reaction catalysed by CALB and conjugation to isoniazid (INH).

Multiple copies of antibiotics can be incorporated into the polymer chain, which becomes active upon pH-triggered hydrolysis to achieve targeted release of a high drug payload. As a proof-of-concept, we incorporated isoniazid (INH) by reacting its hydrazide group with the ketone group of an α-keto polyester (Fig. 1). However, this strategy also allows for the incorporation of other polar antibiotics, such as the first line drug ethambutol or AZD5847 - a next generation oxazolidinone currently in Phase II clinical trials [16], which could be used as the polyol monomer instead of 1,8-octanediol. On the other hand, hydrophobic antibiotics, with poor water solubility and poor caseum penetration [17], can be easily encapsulated in these systems, providing a mechanism of synchronous nanoscale delivery of hydrophilic and hydrophobic payloads, while preventing undesirable drug-drug interactions.

Nanobiotics could be an invaluable tool for delivering drugs in a spatiotemporal-controlled manner, increasing the bioavailability of drugs in the target tissue, while simultaneously protecting drugs against degradation and minimizing their toxic effects in patients.

## 2 Materials and methods

### 2.1 Chemicals

All reagents were analytical grade. 1,8-Octanediol, calcium pantothenate, citric acid, chloroform, clofazimine (CFZ), coumarin 6 (Cou-6), dimethyl 2-oxoglutarate, dimethyl sulfoxide (DMSO) anhydrous, diphenyl ether, Dulbecco’s Modified Eagle’s Medium - high glucose (DMEM), hexane, L-leucine, poly(vinyl alcohol) (PVA; M_w_ 31,000–50,000; 98–99% hydrolysed), phosphate buffered saline tablets, phenol red, phorbol 12-myristate 13-acetate (PMA), silicon oil, sodium phosphate dibasic, Tween 80 and tricaine were purchased from Sigma-Aldrich, Merck (UK). Dichloromethane, formaldehyde, hygromycin B, methanol, Middlebrook 7H9 with OADC, Remel Middlebrook 7H10 Agar (Dehydrated) and trifluoroacetic acid (TFA) were acquired from Thermo Fisher Scientific (UK). Dimethyl sulphoxide-[D₆] (99.8% D), fetal calf serum (FCS; Sera Plus, EU approved regions, special processed FBS, 0.2 μm sterile filtered) and macrophage colony stimulating factor (MCSF) were purchased from VWR (UK), PanBiotech (Germany) and Peprotech (UK), respectively.

### 2.2 Antibodies

The following antibodies were used for flow cytometric studies: Brilliant Violet 785™ anti-human CD3 Antibody (Biolegend®, clone OKT3, 317330), CD14 Monoclonal Antibody APC-eFluor 780 (eBioscience™, clone 61D3, 47-0149-42), PE/Cy7 anti-human CD15 (SSEA-1) antibody (Biolegend®, clone W6D3, 323029), CD19 Monoclonal Antibody PE (eBioscience™, clone HIB19, 12-0199-42). The following isotype controls were used: Mouse IgG1 kappa Isotype Control, APC-eFluor 780 (eBioscience™, clone P3.6.2.8.1, 47-4714-82), Brilliant Violet 785™ Mouse IgG2a, κ Isotype Ctrl Antibody (Biolegend®, MOPC-173, 400273), PE/Cy7 Mouse IgG1, κ Isotype Ctrl Antibody (Biolegend®, MOPC-21, 400125), Mouse IgG1 kappa Isotype Control, PE (eBioscience™, clone P3.6.2.8.1, 12-4714-81). All antibodies were used at 1/250 dilution.

### 2.3 Blank and INH polymer synthesis

1,8-Octanediol was melted in a round bottom flask at 75 °C using a silicon oil bath. Dimethyl 2-oxoglutarate (1:1 M ratio to 1,8-octanediol) and Lipase acrylic resin from *Candida antarctica* (CALB beads; ≥5000 U/g) (10% (w/w) relative to monomers) were added to the flask and the reaction was left for 1 h at 75 °C under vacuum with agitation. Temperature was increased to 90 °C, diphenyl ether (3× volume of monomer) was added and reaction was incubated for another 5 h. After the reaction mixture cooled to room temperature, chloroform (4× volume of monomer) was added to the flask, and the solution was filtered to remove the CALB beads. The crude solution was then precipitated into a 20-fold excess of hexane to remove unreacted monomer. Precipitation was repeated twice, and the obtained copolymer was dried under vacuum overnight. A number average molecular weight (Mn) of 5265 ± 487 g/mol and a dispersity (Ð) of 2.247 ± 0.395 (average of three different polymer batches) were determined for the synthesized polymer (blank polymer) by gel permeation chromatography (Agilent 1260 Infinity II GPC/SEC system). Briefly, a sample of blank polymer was dissolved in chloroform, 0.22 μm-filtered and injected (50 μL) into a PLgel MiniMIX-B column (Agilent). Chloroform was used as eluent at a flow rate of 0.3 mL/min in a 30 min run at 25 °C. The column was calibrated using polystyrene standards (Agilent).

Blank polymer was dissolved in anhydrous DMSO to a final concentration of 200 mg/mL, INH (2 M equivalents excess of keto groups in the polymer) was added and the mixture was kept at 37 °C with orbital agitation for 72 h. After that time, the bright yellow INH-functionalized polymeric solution was added dropwise to methanol (1:10 (v/v)) and subsequently poured into distilled deionised (dd) water (1:2.5 (v/v)) to remove any unreacted INH. The sample was then centrifuged for 1.5 h at 8000 rpm, supernatant was discarded and the polymeric pellet (INH Polymer) was dried overnight under vacuum.

### 2.4 Polymer characterization by Fourier-transform infrared spectroscopy (FTIR)

The chemical fingerprints of INH, blank polymer and INH polymer were determined by FTIR (PerkinElmer Spotlight 400 Frontier FT-IR equipped with Universal ATR) with a scan range of 650-4000 cm^−1^. Data analysis was performed in PerkinElmer Spectrum 10.5.3.

### 2.5 Polymer characterization by ^1^H-NMR

Polymers were dissolved in deuterated DMSO at ∼5 mg/mL concentration. A Bruker Avance III HD 500 MHz equipped with ^1^H/^13^C dual cryoprobe was used to conduct ^1^HNMR measurements. A 10,000 Hz sweep width was observed, acquired using a digital resolution of 64 K points over 3.28 s. A 30° pulse angle was used; based on a 10.5 μs, 14 W pulse at 500.053 MHz being the nominal 90° pulse. 32 scans were accumulated; with an interpulse delay (D1) of 1 s. Data were analysed using Mnova NMR software (Mestrelab Research).

### 2.6 Formulation of nanobiotics

The polymer (either blank polymer or INH polymer) was dissolved in 2 mL of dichloromethane to a final concentration of 10 mg/mL. The polymer solution was added dropwise to 10× volume of an aqueous solution of 1% (w/v) PVA and homogenised for 10 min at 30,000 rpm (VWR Homogenizer VDI12). The emulsion was then probe sonicated for 3 min (35% Amplitude; Pulse: 3 s ON, 6 s OFF), and stirred overnight at room temperature to evaporate dichloromethane. Finally, the sample was centrifuged for 30 min at 8000 rpm and pellet was washed with and resuspended in dd water. For nanobiotics containing CFZ or Cou-6, the compounds were first solubilized in the INH Polymer solution to a final concentration of 5 mg/mL and 0.1 mg/mL of each compound, respectively, and procedure was followed as described above. INH loading was determined by high-performance liquid chromatography (Agilent 1260 Infinity II LC system). Briefly, a sample of nanobiotics was diluted in 1% TFA (v/v) (1:5 or 1:10) and incubated for 48 h at 37 °C, the sample was then centrifuged for 10 min at 14,000 rpm and injected (20 μL) into a Zorbax 300SB C18 column (Agilent). Samples were run for 10 min at 25 °C and flow rate of 0.9 mL/min using an isocratic gradient (0.1% TFA). Absorbance was followed at 260 nm and solutions of known concentrations of free INH were used for calibration. Data analysis was performed using OpenLAB CDS ChemStation for LC 1.15.26 (Agilent). CFZ loading was determined by solubilising the nanobiotics in DMSO and measuring the absorbance at 450 nm in a microplate reader. Solutions of known concentrations of free CFZ were used for calibration.

### 2.7 Nanobiotics characterization by Dynamic Light Scattering (DLS) and Electrophoretic Light Scattering (ELS)

The hydrodynamic size and zeta potential of the nanobiotics were measured by DLS and ELS, respectively, with a Zetasizer Nano ZS system (Malvern Panalytical) fitted with a 4 mW He-Ne laser operating at 633 nm. Measurements were performed at 25 °C and 173° angle at a final nanobiotic concentration of 0.05 mg/mL in dd water. Data were analysed using Zetasizer Software 7.13 (Malvern Panalytical).

### 2.8 Cryo-electron microscopy (Cryo-EM)

All samples were vitrified with a Thermo Fisher Vitrobot MkIV by plunge freezing in liquid ethane. The Vitrobot blot force was calibrated to give a "wedge" of thick ice on roughly 1/3 of the grid, with a gradient of ice thicknesses on the other 2/3 of the grid, corresponding to a setting of "-6" on this system. Other Vitrobot conditions are: temperature 4 °C, RH 100%, blot time 2.5 s, and volume of sample applied 2.5 μL. Quantifoil R1.2/1.3 300 mesh grids were used and made hydrophilic by glow discharge in a weak vacuum in a Pelco Easiglo glow discharge unit at 0.39 mbar for 60 s at 25 mA. Images were acquired on a Thermo Fisher Krios G2 with the single particle data acquisition package EPU (1.10) on a Falcon 3 direct detector at magnifications of 37,000× (2.26 Å/pixel), 47,000× (1.77 Å /pixel), and 6,500× (24.7 Å /pixel). Tomography tilt series +/- 60 deg were acquired with Tomography 4 software on a Falcon 3 detector in counting mode at a nominal magnification of 37,000× corresponding to a total accumulated dose of ∼100 e^−^/Å^2^. Tilt series were aligned by cross-correlation with a stretching factor for tilt and reconstructed by 10 iterations of a Simultaneous Iterative Reconstruction Technique (SIRT) algorithm in Thermo Fisher Inspect 3D 4.3. Visualization and rendering were performed in Thermo Fisher Amira 6.5.

### 2.9 *In vitro* release of INH at different pH

Nano INH were resuspended in 3 different buffers: PBS pH 7.4, citrate-phosphate pH 6 and citrate-phosphate pH 5. The resuspended nanobiotics were aliquoted (100 μL volume; 1.5 mM initial INH concentration) in triplicates and incubated at 37 °C under mild agitation. At pre-defined time intervals, nanobiotic suspensions were centrifuged at 14,000 rpm for 15 min. Supernatant (80 μL) was collected and analysed for drug content by HPLC as described above. Fresh buffer (80 μL) was added and the nanobiotics were resuspended and incubated for another time interval.

### 2.10 Flow cytometry study of nanobiotic uptake by peripheral blood cells

Peripheral blood from healthy volunteers (Regional Ethics approval: REC: 14/EE/1187 IRAS: 161095) was centrifuged at 500 *g* and 21 °C for 10 min. The supernatant (plasma) was discarded and the pellet was resuspended and incubated at 37 °C for 30 min. Nano INH Cou-6 were added to the cells (to a nanobiotic final concentration of 0.05 mg/mL) and the mixture was incubated at 37 °C for 30 min, followed by 15 min at 4 °C. Clinical grade polyclonal human IgG (Vivaglobin ®) was added to the cells and incubated at 4 °C for 5 min to block Fc receptors. Cells were stained with the antibodies above for 30 min at 4 °C. Red blood cells were lysed using BD FACS™ lysing solution (BD Biosciences), fixed with BD Cell Fix and transferred to Corning™ Falcon™ test tube with cell strainer snap cap. Samples were analysed using an BD LSRFortessa™ cell analyzer (BD Biosciences). Data were processed using FlowJo® 10.5.0 software (FlowJo LLC).

### 2.11 *In vitro* mycobacterial infection assays

Primary monocyte-derived human macrophages, generated as described [18], from healthy consented subjects (Regional Ethics approval: REC: 14/EE/1187 IRAS: 161095), or THP-1 cells (ATCC) were differentiated by treatment with either 100 ng/mL MCSF or 5 ng/mL PMA 48 h before infection, inoculated with *M. tuberculosis H37Rv* ΔleuD ΔpanCD (Bleupan) [19], grown as described [20], using a multiplicity of infection (MOI) of 10:1 for 2 h at 37 °C, washed with PBS, and incubated with either DMEM media, supplemented with 10% FCS, 0.4% L-leucine, 0.1% calcium pantothenate (untreated control), Nano Blank in media (negative control), INH in media (positive control) or Nano INH in media for 48 h at 37 °C. Two drug concentrations were tested: 10 μM and 100 μM. Cells were lysed by osmotic shock and intracellular bacteria were counted. In case of primary monocyte-derived human macrophages, cells were lysed and plated to count colony-forming units (CFUs). In the case of THP-1 cells, a validated luminescent reporter strain of *M. tuberculosis H37Rv* ΔleuD ΔpanCD (Bleupan) [19] encoding the Vibrio *luxAB* gene was used for infection and luminescence was measured as described [18] after cells lysis. Correlation between CFUs and luminescence was established before experiments. Experiments were carried out in sextuplicate.

### 2.12 *In vitro* nanobiotics uptake by THP-1 cells

THP-1 cells were plated on glass coverslips, infected with a mCherry fluorescent reporter strain of *M. tuberculosis H37Rv* ΔleuD ΔpanCD (Bleupan) [19] using a MOI of 10:1, and treated for 1 h with Nano INH Cou-6 (to a final concentration of 50 nM Cou-6), rinsed with PBS, fixed with 4% formaldehyde, rinsed with water and then mounted with ProLong Gold antifade containing DAPI (Invitrogen). Images were acquired on a Zeiss 780 confocal microscope (Plan-Apochromat × 63/1.40 Oil-immersion lens) and analysed with Zen 2010 (Carl Zeiss) and Fiji (open source).

### 2.13 Zebrafish husbandry and ethic statements

Experimental procedures were performed using the nacre line zebrafish. Transgenic *Tg(mpeg:mCherryCAAX)sh378* zebrafish line was used to visualize macrophages chemotaxis towards injection sites. Zebrafish were raised and maintained according to standard protocols in UK Home Office-approved facilities in The Bateson Centre aquaria at the University of Sheffield under AWERB (Animal Welfare and Ethical Review Bodies). Eggs were obtained from pairs of adult fish by natural spawning and raised at 28.5 °C in tank water. All animal experiments described in the present study were conducted on the Project Licence P1A4A7A5E held by Professor Stephen Renshaw at the University of Sheffield.

### 2.14 *In vivo* mycobacterial infection and treatment

*M. marinum* strain M carrying pTEC27 (Addgene, plasmid 30182) that express red fluorescent protein (tdTomato) were grown at 28.5 °C under hygromycin B selection in Middlebrook 7H9 broth medium supplemented with oleic acid, albumin, dextrose, catalase (OADC) enrichment and 0.05% Tween 80 (7H9^OADC/T^). Mid‐log‐phase cultures of *M. marinum* expressing tdTomato were pelleted, washed twice and resuspended in PBS Tween (PBST). Mycobacterial suspensions were then homogenized through a 26‐gauge needle and adjusted to an optical density at 600 nm (OD_600_) of 1 in PBST and mixed with phenol red.

Microinjections of 2 nL of bacterial suspensions of known concentration (containing around 150 mycobacteria) were carried out directly into the caudal vein in 30 hpf embryos previously dechorionated and 0.02% w/v tricaine-anesthetized. The inoculum size was checked by injection of 2 nL in sterile PBST and plated on 7H10^OADC^ agar. Infected embryos were then transferred into plates and incubated at 28.5 °C.

At 4 h post-infection, either free antibiotics or nanobiotics of known concentrations were intravenously administered to embryos. Groups of infected/treated embryos were then transferred into 6‐well plates and incubated at 28.5 °C. To determine efficiency of nanodrugs *vs* free drugs on infection outcomes, embryos were collected at 3 days post infection/treatment and imaged for both granuloma quantification (defined at least 10 infected cells) and bacterial burdens analysis as Fluorescent Pixel Count (FPC) by fluorescence microscopy.

### 2.15 Macrophages recruitment observation

Macrophage mobilization towards nanobiotic-injected sites was elicited through injection of Cou-6-labelled Nano Blank into the muscle compartment of 3 days post-fertilization transgenic larvae *Tg(mpeg1:mCherry-CAAX)*sh378 [21]. Leucocytes chemotaxis was visualized and imaged at 1 and 4 h post-injection using confocal microscopy.

### 2.16 Epifluorescence, confocal microscopy and imaging

Epifluorescence microscopy was performed using a Leica MZ10 F stereomicroscope (Leica Microsystems, Germany) equipped with GXCAM-U3 Series 5 M P (GT Vision) camera. Confocal microscopy was performed using a Leica TCS-SPE confocal DMi8 inverted microscope (Leica Microsystems, Germany) using a HC FL PLAB 10x/0.40, 20x or 40x objective lenses and captured using a Hammamatsu ORCA-Flash 4.0 camera (Hammamatsu, Japan).

### 2.17 Statistical analysis

All data are expressed as mean ± SEM. Statistical analysis for comparing two experimental groups was performed using two-sided Student’s t-tests. A value of P < 0.05 was considered statistically significant. Analyses were performed with Prism 7 (Graph pad Software). Differences are labelled n.s. for not significant, * for P ≤ 0.05, ** for P ≤ 0.01, *** for P ≤ 0.001 and **** for P ≤ 0.0001. The sample size of each experiment was determined to be the minimal necessary for statistical significance by the common practice in the field. No animals were excluded from the experiments.

## 3 Results and discussion

### 3.1 Synthesis and characterization of isoniazid-based polymer

**Fig. 2:**
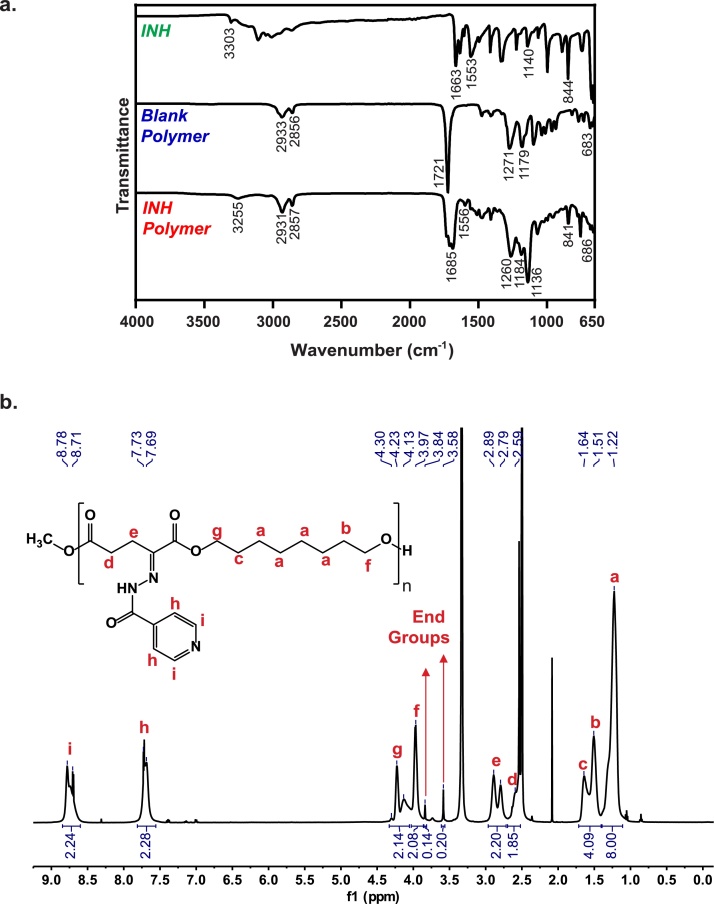
Characterization of polymer-drug conjugates. **a**. FTIR spectra of INH (top), Blank Polymer (middle), and INH Polymer (Bottom). **b**. ^1^H-NMR spectrum of INH Polymer in DMSO-*d*_6_ and peak assignments.

Both blank and INH-based polymers were characterized by Fourier-transformed infrared spectroscopy (FTIR) (Fig. 2**a**) and ^1^H nuclear magnetic resonance (NMR; Fig. 2**b**). The characteristic FTIR peaks from the α-keto polyester blank polymer appear at 2933 and 2856 cm^−1^ due to C–H stretching vibrations from CH_2_ and CH_3_ functional groups, at 1271 and 1179 cm^−1^ due to C–O stretching from ester groups, and at 683 cm^−1^ due to C—C=O bending. The INH drug has a characteristic peak at 3303 cm^−1^, related to N—H stretching of the hydrazide group, which shifts to 3255 cm^−1^ and reduces in intensity upon formation of the hydrazone bond during conjugation to the polymer [22]. In addition, INH-polymer conjugation also generates peaks at 1556 cm^−1^ (from H-N-N bending), at 1136 cm^−1^ (from N—N stretching of the hydrazide group), and at 841 cm^−1^ (from ring C—C—H bending vibrations). The peaks in the region of 3100-2900 cm^−1^ characteristic from C–H stretching vibrations of heteroaromatic compounds, present in the INH spectrum, are too weak to observe following polymer incorporation. Bands at 3105 cm^−1^, 1633 cm^−1^ and 1321 cm^−1^ generated from stretching and bending vibrations of the NH_2_ group are present in the INH spectrum, disappear from the INH polymer spectrum, corroborating the formation of the hydrazone bond. The peaks at 1663 cm^−1^ (for INH), 1721 cm^−1^ (for the blank polymer), and 1685 cm^−1^ (for the INH polymer) correspond to C=O stretching of several carbonyl groups.

The ^1^H-NMR spectra of the blank (**Figure S1;** chemical shifts reported in ***Supplementary Information***) and INH (Fig. 2**b;** chemical shifts reported in ***Supplementary Information***) polymers in DMSO-*d*_6_ confirmed our predicted structure. The spectrum of the blank polymer shows the presence of peaks at 2.53–2.56 ppm and 3.04–3.07 ppm from the protons of the ketoglutarate unit and peaks between 1.26 ppm and 1.67 ppm attributed to the aliphatic protons of the octanediol unit. Protons α to the backbone ester group on the octanediol unit produced peaks at 3.97–4.00 ppm and 4.16–4.19 ppm (**Figure S1)**. The INH polymer spectrum shows new peaks at 7.69–7.73 and 8.71–8.78 ppm attributed to the aromatic protons of INH. The integration ratio of INH aromatic protons to polymer chain protons indicated complete conjugation of the INH to the polymer. The integration ratio of protons from the methyl ester end-groups to protons on the repeating unit indicates a polymer chain with an average of 16 and 18 repeating units, equivalent to a Mn of 4147 g/mol for the blank polymer and a Mn of 6804 g/mol for INH polymer, respectively.

### 3.2 Formulation and characterization of nanobiotics and *in vitro* drug release

**Fig. 3:**
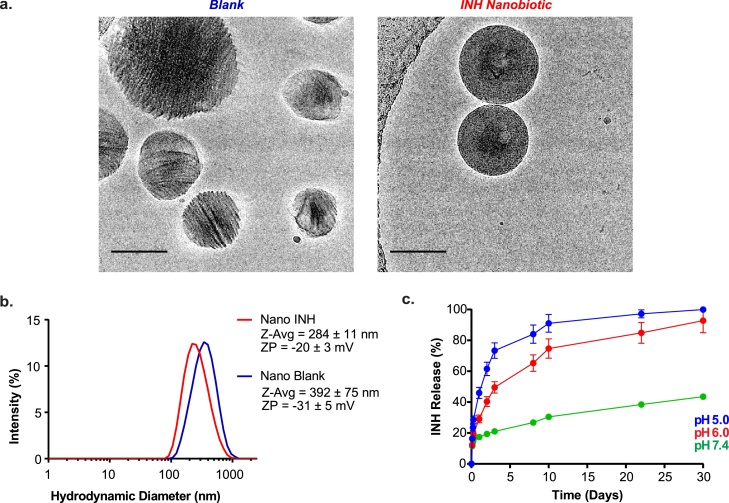
Characterization of polymeric nanobiotics **a**. Cryo-EM images show different shape and crystallinity of the Blank (left) and INH (right) nanobiotics (scale bar, 100 nm). **b**. Size distribution (nm) and zeta potential (mV) of Blank (blue) and INH (red) polymeric nanobiotics formulated by single emulsion solvent evaporation technique (n = 3). **c.** INH release from Nano INH at pH 5 (blue), 6 (red), and 7.4 (green), mimicking the acidic conditions of the phagolysosome and physiological conditions of systemic circulation (n = 3). (For interpretation of the references to colour in this figure legend, the reader is referred to the web version of this article).

Nanoparticles were generated from both blank and INH-conjugated polymers using single-emulsion solvent evaporation [23], which we visualized in their native solutions using Cryo-EM [24,25] (Fig. 3**a**). Nanoparticles formulated from blank polymer were irregular in shape and presented crystalline visual appearance with regular shaded patterns (consistent with the semi-crystalline nature of many polyesters [26]) while those made from INH polymer were perfectly spherical (reportedly favouring uptake by phagocytes [27]), densely packed, and presented amorphous visual appearance, possibly due to an increase in disordered regions due to the presence of the bulky pyridyl ring after drug conjugation [28]. Using Dynamic Light Scattering (Fig. 3**b**), we found that, compared to blank nanoparticles, INH nanobiotics were slightly smaller (Z-average of 284 ± 11 nm compared to 392 ± 75 nm), had similar polydispersity index (0.321 compared to 0.344), and had less negative zeta potentials (-20 ± 3 mV compared to -31 ± 5 mV; consistent with the presence of basic functional groups). INH loading in the nanobiotics was 25 ± 5% wt (g INH/g nanobiotic), higher than 16% wt reported for INH-chitosan conjugates [13] and 30 times higher than the 0.8% wt (7.78 mg/g) reported for other INH conjugated polyesters, such as PLGA [15].

We next examined the drug release properties of INH nanobiotics *in vitro* (Fig. 3**c**). As expected from the hydrolytic cleavage of the hydrazone bond, greater and more rapid INH release was observed during incubation at acidic pH levels that replicate those reported for *Mt*b-containing phagosomes [29] and the inside of *Mtb* granulomas [29,30].

### 3.3 *In vitro* evaluation of INH nanobiotics in *Mtb*-infected human primary macrophages and THP-1 cells

We hypothesized that nanobiotics would be successfully targeted to infecting *Mtb* since, following internalization by phagocytes, they would be targeted directly to intracellular mycobacteria, through endosomal fusion [31], and delivered to extracellular mycobacteria in granuloma or cords through the frequent trafficking of macrophages and neutrophils to these sites [[32], [33], [34]].

**Fig. 4:**
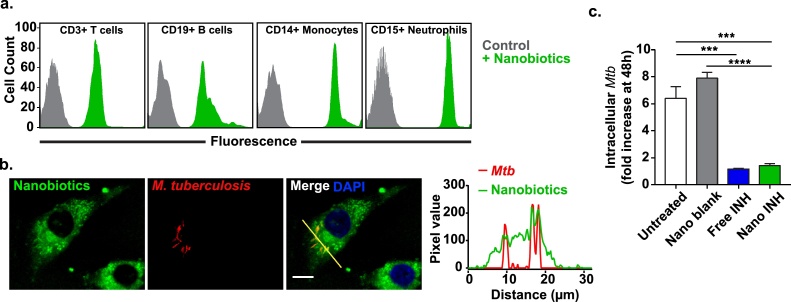
Nanobiotic uptake by phagocytic cells and *in vitro* efficacy against *M. tuberculosis.***a**. Nanobiotics uptake by white blood cells quantified by fluorescence-activated cell sorting (FACS). Coumarin 6-labelled Nano INH are preferentially uptaken by phagocytic cells, namely monocytes (CD14+) and neutrophils (CD15+), rather than lymphocytes, such as T cells (CD3+) and B cells (CD19+), likely reflecting their large size distribution. **b**. Confocal microscopy images of differentiated THP-1 cells infected with a mCherry fluorescent reporter strain of *M. tuberculosis H37Rv* ΔleuD ΔpanCD (red) and treated with Cou-6-labelled Nano INH (green) (scale bar, 10 μm) **c**. Differentiated THP-1 cells were infected with a luminescent reporter strain *M. tuberculosis H37Rv* ΔleuD ΔpanCD, treated with 100 μM INH either as a free drug or as nanodrug and intracellular *M. tuberculosis* was assessed 48 h post-infection by relative luminescence units (RLUs). Untreated cells and cells treated with drug-free nanobiotics (Nano Blank) were used as negative controls. Results are presented in terms of RLUs normalized to untreated cells (Mean ± SEM, n = 6). (For interpretation of the references to colour in this figure legend, the reader is referred to the web version of this article).

To investigate uptake by human cells, we incubated peripheral blood samples from healthy subjects with fluorescently-labelled INH nanobiotics. Using flow cytometry, we observed rapid high-level accumulation in all monocytes and neutrophils, as well as less efficient uptake by B and T cells (Fig. 4**a**). We also monitored internalization by *Mt*b-infected cells using confocal microscopy (Fig. 4**b; *Supplementary Movie***). Nanobiotics were avidly taken up by macrophage cell lines and trafficked to internal compartments including mycobacteria-containing phagosomes. We then confirmed that INH nanobiotics were active against intracellular *Mtb,* showing equivalent potency to free INH when added to infected THP-1 cells and primary human macrophages (Fig. 4**c; *Supplementary Figure S2***).

### 3.4 Pre-clinical studies in a zebrafish larval model of mycobacterial infection

**Fig. 5:**
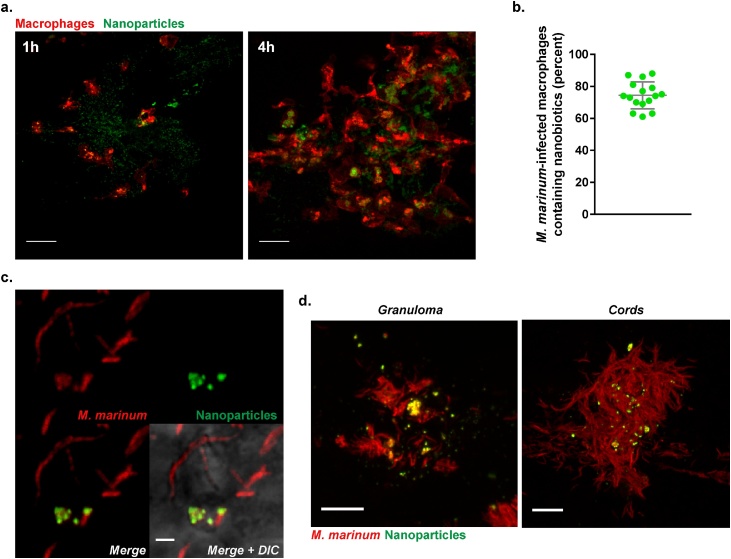
*In vivo* testing of multi-drug nanobiotics in a *M. marinum*-infected zebrafish larval model. **a**. Confocal microscopy images showing nanobiotics-induced macrophage mobilization *in vivo*. Suspension of coumarin 6-labelled Nano Blank (green) was injected into the muscle of 3 dpf *Tg(mpeg1:mCherryCAAX)sh378* zebrafish line harbouring red macrophages. Macrophage chemotaxis towards injection site has been monitored at 1 and 4 h post injection (scale bar, 20 μm). **b.** Quantification and **c.** Confocal imaging of coumarin 6-labelled Nano Blank (green) uptake by *M. marinum*-infected macrophages (red) after 4 h post infection (scale bar, 1 μm). **d.** Confocal imaging showing the repartition and accumulation of coumarin 6-labelled Nano Blank (green) into a *M. marinum (red)-granuloma* (left, scale bar, 5 μm) and a mycobacterial cord structure (right, scale bar, 5 μm). (For interpretation of the references to colour in this figure legend, the reader is referred to the web version of this article).

We proceeded to examine the fate and activity of nanobiotics *in vivo* by exploiting the optical transparency of zebrafish larvae. Nanoparticles were rapidly engulfed by macrophages following intramuscular injection (Fig. 5**a**) and, in fish infected with *M. marinum* (a pathogenic mycobacterial species closely related to *Mtb*), nanoparticles were taken up by over 70% of all infected macrophages (Fig. 5**b**&**c**). By 3 days post infection, we observed delivery of nanoparticles, presumably by macrophages, to both granuloma and extracellular mycobacterial cords (Fig. 5**d**).

We next explored the potential application of nanobiotics for synchronous delivery of multiple drugs and successfully encapsulated CFZ within INH-nanobiotics (Nano INH & CFZ), with a drug loading of 22 ± 1% wt (g CFZ/g nanobiotic), and these remained structurally stable in solution for 9 months at room temperature (***Supplementary Figure S3***).

**Fig. 6:**
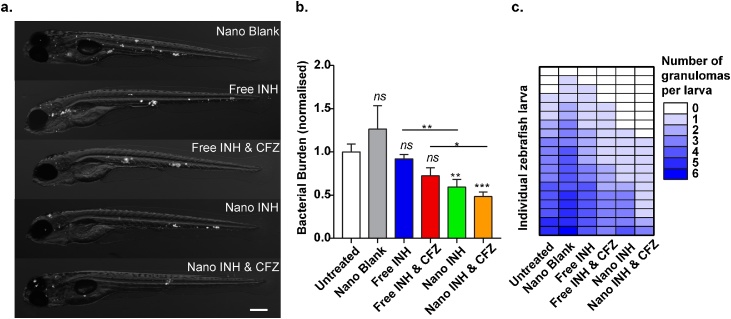
Effect of nanobiotics at 3 days post infection on zebrafish infected with fluorescently-labelled *M. marinum.***a**. representative images (scale bar, 200 μm). **b**. quantification of bacterial load (results plotted as mean ± SEM from 2 independent experiments; n = 21). **c**. Quantification of granuloma number at 3dpi. Results are plotted as mean ± SEM from 2 independent experiments (n = 19).

Zebrafish larvae were then infected with *M. marinum* and, 4 h later, treated with either free drug (INH alone or INH with CFZ) or injected with nanobiotics (Nano INH, Nano INH & CFZ, or blank nanoparticles). At 3 days post infection, both INH- and INH & CFZ- nanobiotics, but not the equivalent concentration of free drugs, were able to significantly reduce bacterial burden and granuloma number in *M. marinum-*infected fish compared to controls (Fig. 6**a**-**c**).

Due to the wide-ranging tools and strains available, the mouse infection model has been the most extensively studied in *Mtb* research. However, the main disadvantage of this model is the inability of mice to effectively replicate human pathologies, such as the caseous granuloma formation. Instead, mice form diffuse and noncaseating lesions, likely due to the fact that *Mtb* is not a natural pathogen of mice. Other mammalian models, such as guinea pigs and rabbits, which produce necrotic granulomas and more closely resemble the human *Mtb* pathology, are not as amenable for transgenic and knockout line production. The primate infection model (*e.g.* macaques) is perhaps the most clinically relevant, but it is limited by high costs and ethical restrictions [35,36].

In recent years, zebrafish has been recognised as a useful vertebrate animal model, particularly due to its low cost, ease of manipulation and optical transparency, which allows non-invasive and real-time monitoring using imaging tools of host-pathogen interactions at a cellular level in a live animal [37]. Despite mammal models being evolutionary more similar to humans, zebrafish and human genomes present high homology (71% of human protein-encoding genes and 82% of disease related genes have zebrafish orthologues), with functional domains of proteins being almost identical in both species [38]. The zebrafish-*M. marinum* model also presents pharmacological similarities (*i.e.* similar effect of drugs) and homologous immune responses to humans, including robust granuloma formation [35,36]. This model is not, of course, intended to replace mammalian infection models, but is rather an unique and powerful tool for phenotypic screenings and to study pathophysiological events.

## 4 Conclusions

We report a smart multi-drug delivery vehicle, which allows the simultaneous incorporation of both hydrophilic and hydrophobic drugs at high concentrations and their targeted delivery to both intracellular and granuloma-resident mycobacteria *in vivo*. The main advantage of this system is the synthetic simplicity and versatility. The drug is directly conjugated to the polymer without the need for any further chemical modifications. The drug-polymer bond is acid-labile, allowing site-specific drug release, and the polymer itself is hydrolysable facilitating excretion. Polymer size can be tuned without affecting the high drug loading capacity, since there is one drug conjugation site per monomeric unit of polymer. With the slow development of new antibiotics, tunable polymeric nanobiotics have the potential to deliver more effective and more tolerable combination chemotherapy using existing drugs for *Mtb* and other infectious diseases.

## Funding

This work was supported by the Rosetrees Trust Interdisciplinary Prize 2015 (ILB, MS, MEW, RAF), the Wellcome Trust awards 107032/Z/15/Z (RAF) and 10/H0305/55 (MS), the NIHR Cambridge Biomedical Research Centre Award (RAF), the MRC AMR Theme award MR/N02995X/1 (SAR, AB) and Marie-Curie IF CFZEBRA 751977 (AB).

## Author contributions

I.L.B. designed and performed the experiments, and analysed the data. A.B. and S.A.R. were responsible for the *in vivo* testing in zebrafish model. C.P. assisted with bacterial killing assays. C.K. assisted with fluorescence-activated cell sorting. K.S. and P.C.H. performed cryo-EM imaging. M.S., M.M.O., S.K. and S.M. started the project and performed initial experiments. I.L.B., R.A.F. and M.E.W. wrote the paper, with contributions from all co-authors. R.A.F. and M.E.W. supervised the project.

## Declaration of Competing Interest

The authors declare no conflict of interest.
