# Supplementary material for: Polymeric nanobiotics as a novel treatment for mycobacterial infections
Source: J Control Release. 2019 Nov 28;314:116–24. doi: 10.1016/j.jconrel.2019.10.009 (PMC6899522; doi:10.1016/j.jconrel.2019.10.009)
Supplement: Supplementary file 1 [file mmc1.docx]

**Supplementary Information**

**NMR Chemical Shifts**

**Blank Polymer**

^1^H-NMR (500 MHz, DMSO-d6): δ 4.17 (t, J = 15 Hz, 2H), δ 3.98 (t, J = 15 Hz, 2H), δ 3.06 (t, J = 15 Hz, 2H), δ 2.55 (t, J = 15 Hz, 2H), δ 1.65 (t, J = 15 Hz, 2H), δ 1.54 (t, J = 15 Hz, 2H), δ 1.31-1.26 (m, 8H)

**INH Polymer**

^1^H-NMR (500 MHz, DMSO-d6): δ 8.78-8.71 (br, 2H), δ 7.73-7.69 (br, 2H), δ 4.30-4.13 (br, 2H), δ 3.97 (br, 2H), δ 2.89-2.79 (br, 2H), δ 2.59 (br, 2H), δ 1.64 (br, 2H), δ 1.51 (br, 2H), δ 1.22 (br, 8H)


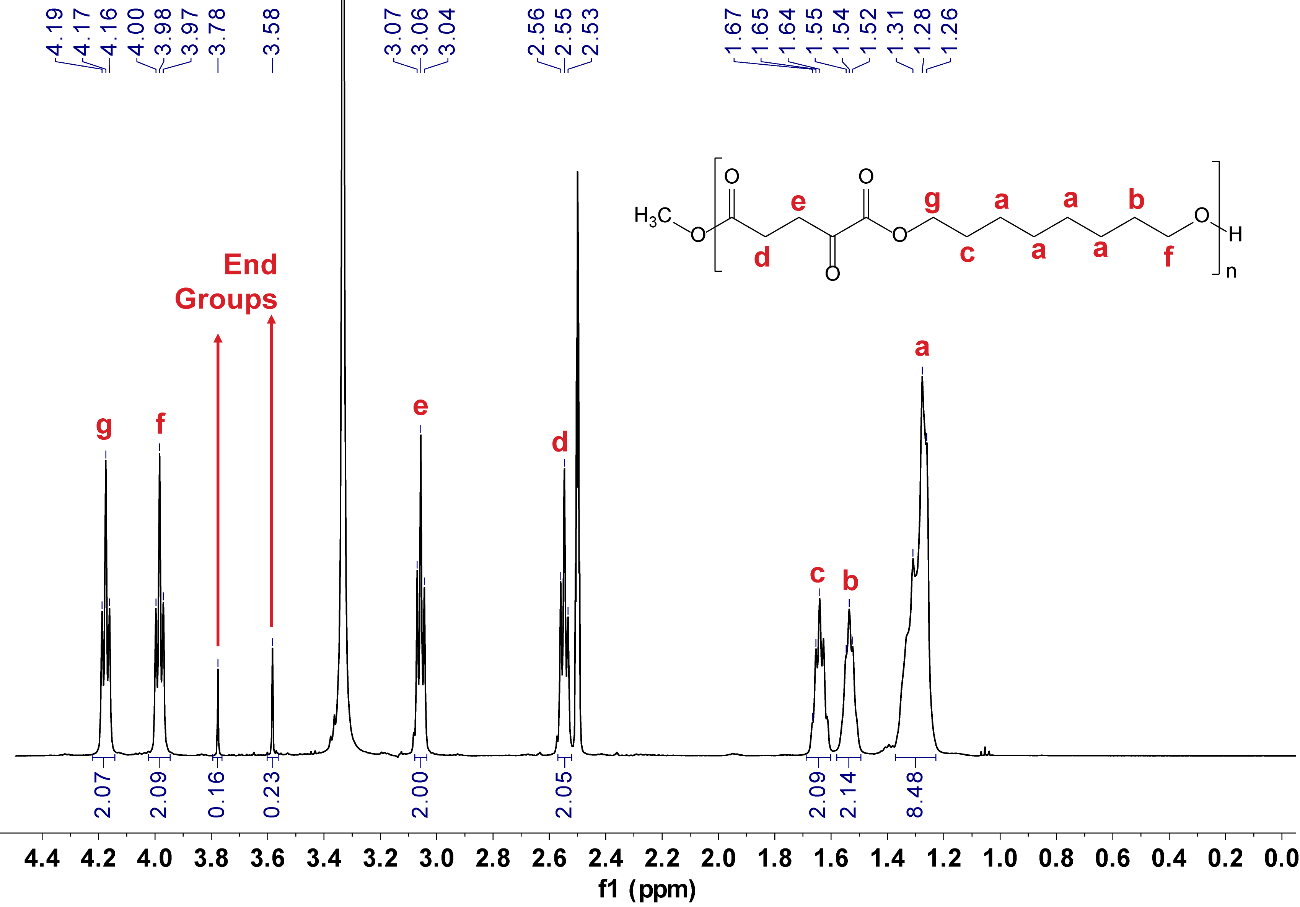


**Figure S1.** ^1^H-NMR spectrum of Blank Polymer in DMSO-d6 and peak assignments.

**
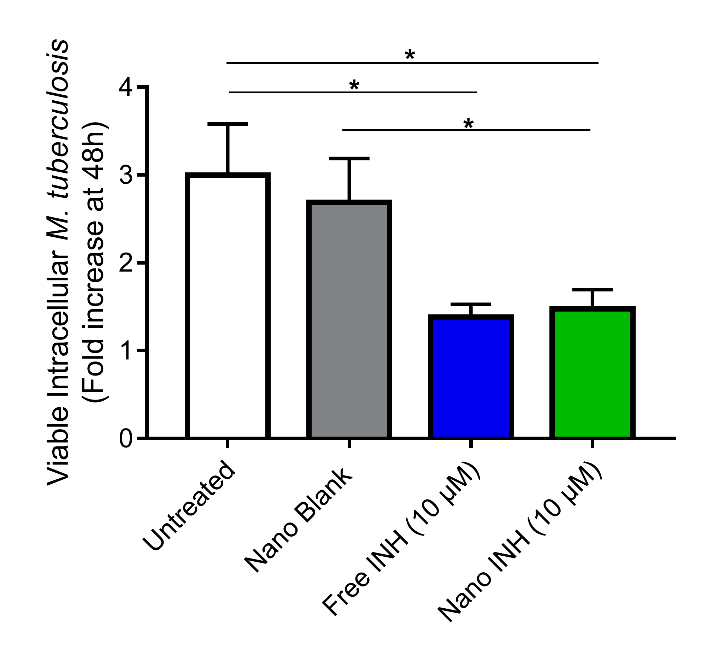
**

**Figure S2.** Human primary macrophages were infected with *M. tuberculosis* *H37Rv* ΔleuD ΔpanCD, treated with 10 µM INH either as a free drug or as nanodrug and viable intracellular *M. tuberculosis* was assessed 48h post-infection by colony-forming units (CFUs). Untreated cells and cells treated with drug-free nanobiotics (Nano Blank) were used as negative controls. Results are presented in terms of CFUs normalized to untreated cells (Mean ± SEM, n = 6).

**
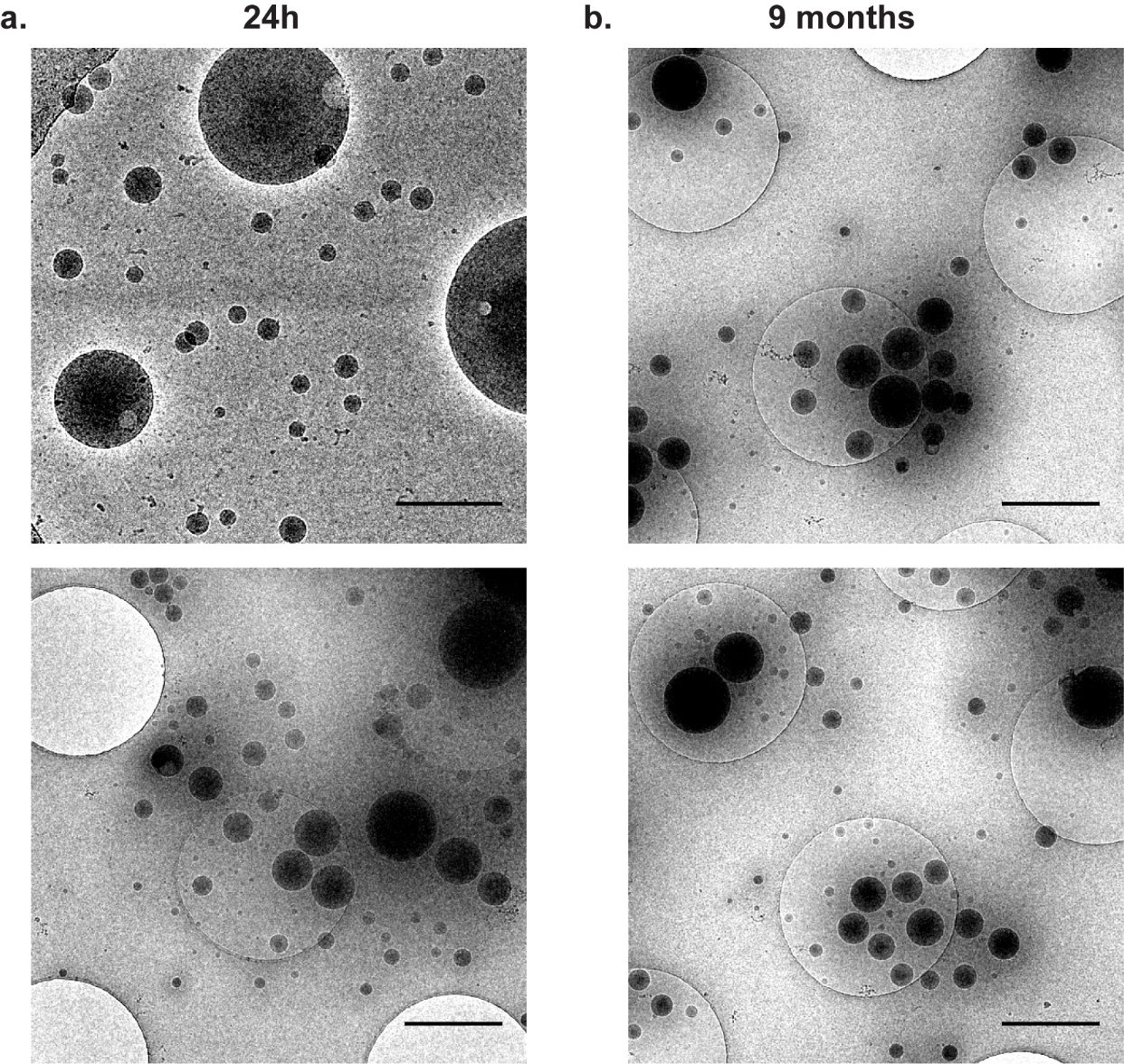
**

**Figure S3.** Cryo-EM images of freshly synthesized (a) and 9 months old (b) INH & CFZ nanobiotics stored at room temperature show the long-term physical stability of the nanobiotics (scale bar, 200 nm for top left image and 1 µm for all others).
